# Sulfuric Acid Assisted Preparation of Red-Emitting Carbonized Polymer Dots and the Application of Bio-Imaging

**DOI:** 10.1186/s11671-018-2657-4

**Published:** 2018-09-10

**Authors:** Chunlin Tan, Chao Zhou, Xingyun Peng, Huozhen Zhi, Dan Wang, Qiuqiang Zhan, Sailing He

**Affiliations:** 1 Centre for Optical and Electromagnetic Research, Guangdong Provincial Key Laboratory of Optical Information Materials and Technology, South China Academy of Advanced Optoelectronics, South China Normal University, Guangzhou, 510006 China; 2 JORCEP, Department of Electromagnetic Engineering, Royal Institute of Technology, 10044 Stockholm, Sweden; 3 Engineering Research Center of MTEES (Ministry of Education), School of Chemistry and Environment, South China Normal University, Guangzhou, 510006 China; 4 State Key Laboratory of Organic-Inorganic Composites, Beijing University of Chemical Technology, Beijing, 100029 China

**Keywords:** Carbon dots, Carbonized polymer dots, Red-emitting, Fluorescence, Sulfuric acid assisted, Hydrothermal

## Abstract

Red-emitting carbonized polymer dots (CPDs) was prepared from *p*-phenylenediamine (*p*-PD) aqueous solution with the assistance of sulfuric acid (H_2_SO_4_), and the optical properties and bio-imaging application were studied in this paper. Compared with other strong acids-assisted systems, SA-CPDs (prepared from H_2_SO_4_-assisted system, average diameter is ~ 5 nm) is the brightest. The photoluminescence Quantum Yields (QYs) is 21.4% (in water), and the product yield is 16.5%. SA-CPDs aqueous solution emits at 600 nm when excited by the light from 300 to 580 nm. The emission wavelength is independent on the excitation wavelength. Formation energies of CPDs in two ways were calculated to show that longitudinal growth (forming polymers) is difficult, and the transverse growth (forming CPDs) is easy. In addition, the two-photon photoluminescence properties (emitting at 602 nm when excited by 850 nm femtosecond pulse laser) of SA-CPDs were also utilized in the experiments for HeLa cells staining and shown to have potential applications in bio-imaging.

**Electronic supplementary material:**

The online version of this article (10.1186/s11671-018-2657-4) contains supplementary material, which is available to authorized users.

## Background

Carbon dots (CDs) have attracted much attention due to their advantages including excellent water solubility, optical stability, unique fluorescence properties, low toxicity, low cost, etc [1]. Most CDs were studied as potential candidates for various applications, such as electrochemical immune-sensors [2], bio-imaging [3–6] sensors [7–12], photo-catalysis [13–15], light-emitting devices [16], and optoelectronics [17–19]. Synthesis of CDs plays an important role in the studies on the optical properties and applications. The reported approaches to prepare CDs could be mainly summarized as the “top-down” from various carbon materials and “bottom-up” from organic molecules, polymers, or natural products [20]. The “bottom-up” methods are efficient routes for the synthesis of fluorescent CDs in large scale [21]. Groups in the applied molecules including –OH, –COOH, –C=O, and –NH_2_ can be dehydrated and carbonized in elevated temperature by hydrothermal, microwave, combustion, pyrolysis, and so on.

Red-emitting dots have aroused considerable interests because of, i.e., the larger penetration depth in bio-imaging field. Especially, the pure color dots are critical to a certain occasion because the excitation-wavelength-independent luminescence materials can provide a single and stable photoluminescence (PL) light. Most emissions of CDs are excitation-wavelength-dependent, and CDs usually emit blue, green, or yellow light, few CDs emit bright red light [22].

Recently, isomers of phenylenediamine (PD), such as *o*-, *m*-, and *p*-PD, had been studied as carbon sources to prepare CDs [8, 9, 23, 24]. Blue-, green-, and red-emitting CDs can be prepared from *m*-, *o*-, and *p*-PD ethanol solution, respectively [23]. Full-color light-emitting CDs can be prepared from *p*-PD and urea aqueous solution [24]. In our previous work [25], we proposed that new red carbon dots (Quantum yields = 15.8%, in water) can be synthesized facilely from “*p*-PD + HNO_3_” aqueous system and applied in the detection of metal ions in water. Recently, the similar “*o*-PD + H_3_PO_4_” [26] and “*o*-PD + HNO_3_” [27] systems were reported, and Liu et al. [27] renamed their CDs (QYs = 10.8%, in water) as “carbonized polymer dots (CPDs).” Unlike the traditional carbon dots, the CPDs’ emission wavelengths do not depend on the excitation wavelength, and thus the PD-based “CDs” should be named more accurately as CPDs.

Herein, we report a facile and high-efficient method of strong acid-assisted hydrothermal route to prepare red-emitting CPDs and the application of bio-imaging with two-photon photoluminescence properties. Mechanism for the formation of CPDs is proposed by using Gaussian 09 program package.

## Methods

### Synthesis of Red CPDs From Acid-Assisted *p*-PD Systems

Based on our previous work [25], we selected sulfuric acid (H_2_SO_4_), hydrochloric acid (HCl), and perchloric acid (HClO_4_) as the assistants for the preparation of red CPDs, the corresponding CPDs were labeled as SA-CPDs, HC-CPDs, and PA-CPDs, respectively. In order to optimize the experimental conditions of H_2_SO_4_-assisted system, we selected several parameters, such as *c* (acid) to *c* (*p*-PD) ratio, *c* (*p*-PD), temperature (*T*), and reaction time (*t*). CPDs products were washed by hexane to remove the unreacted *p*-PD and by ethyl alcohol to remove acids, centrifuged at 14000 rpm for 30 min to remove polymer precipitation, and filtered through a 0.22 μm filter membrane. If powder is desirable, the purified CPDs solution can be further evaporated by rotary evaporator to near dry state at 80 °C and a low vacuum condition (the remaining will be of powder form).

### Characterization and Measurement

High-resolution TEM (HR-TEM) images were recorded on JEM-2100 transmission microscope operating at 200 kV. Infrared spectra of CPDs solutions were collected using Prestige-21 FT-IR spectrometer by use of KRS-5 window slices (mixture of TlBr and TlI), typically, liquid phases were dropped on one slice and dried. The slice was covered by the other slice and fixed on the testing stand. Then the infrared spectra were recorded.

The fluorescence spectra of CPDs were measured on F-2500 fluorescence spectrophotometer. UV-Vis absorption spectra were recorded on Lambda 950 UV/VIS/NIR Spectrometer. The two-photon emission spectra of CPDs were recorded by a fiber spectrograph (QE65000, Ocean Optics) in the microscope system. SA-CPDs aqueous solution and the powders re-dissolved solution were spun on slides, and the two-photon photoluminescence properties were then measured.

Photoluminescence quantum yields (QYs) of the CPDs were measured with Rhodamine B (QYs = 56% in ethanol) as the reference dye at the emission range of 580–610 nm excited by 365 nm UV light [25, 28], the procedure of QYs measurements were shown in the Additional file 1.

### Calculation Methods

The Gaussian 09 package was used for the density function theory (DFT) calculations [29]. The equilibrium structures were optimized by B3LYP method in conjunction with the 6–311++G (d) basis set level [30]. To investigate the role of solvent effects, water was utilized in polarized continuum model (PCM). Frequency analyses were done with the same level for confirming that each optimized structure corresponded to a stationary point.

### Cell Culture and Treatment

1.35 mL of HeLa cells in Dulbecco’s Modified Eagle Medium (DMEM; Gibco) at an initial density of 4 × 10^4^ cell per milliliters were seeded in each dish and cultured at 37 °C for 24 h under a humidified atmosphere containing 5% CO_2_. SA-CPDs powders were re-dissolved in water to prepare the reserve solution (400 μg mL^− 1^). 1350 μL cells were cultured with 150 μL SA-CPDs reserve solution (the final concentration is 40 μg mL^− 1^) for 12 h and then washed three times with PBS to remove the free SA-CPDs. Finally, the cellular imaging results were collected with a confocal microscope under 850-nm femtosecond laser excitation (30 mW).

## Results and Discussion

### Optimizing Preparation for Red CPDs

In basic experiments, different acid-assisted systems with various concentration ratios, reaction temperatures, and times were investigated (see Additional file 1: Figure S1). We found that red CPDs can be formed above 180 °C (reacting for 2 h) for different acids systems, and the reactions are not affected by anions in the solutions. Long-time (4–12 h, 240 °C for H_3_PO_4_ and HF systems, see Additional file 1: Figure S1f) reaction will increase the particle size, and the red fluorescence will be eventually faded, while the fluorescence change is not obvious for HCl system (2–6 h, 200 °C, see Additional file 1: Figure S2). Considering the energy saving and upper temperature limit of Teflon liner, optimum temperature and reaction time are selected as 200 °C and 2 h respectively. Based on the optimization strategy of *p*-PD + HCl system (see Additional file 1: Figure S2), we optimized the *p*-PD + H_2_SO_4_ and *p*-PD + HClO_4_ systems and obtained the optimization results shown as Table 1.

**Table 1:** The selected experimental parameters of *p*-PD + acids systems

System	*c* (*p*-PD) (M)	*c* (acid) (M)	*T* (°C)	*t* (h)
*p*-PD + H_2_SO_4_	0.1	0.1	200	2
*p*-PD + HCl	0.3	0.1	200	2
*p*-PD + HClO_4_	0.3	0.1	200	2

SA-CPDs, HC-CPDs, and PA-CPDs were prepared from *p*-PD solution with the assistance of H_2_SO_4_, HCl, and HClO_4_, respectively. The optimized *c* (acid) to *c* (*p*-PD) ratios of H_2_SO_4_-, HCl-, and HClO_4_-assisted systems are 1, 3, and 3, respectively (see Additional file 1: Figure S3a). The suitable *c* (*p*-PD) range for the preparation of red CPDs is wide (from 0.02 to 0.20 mol L^− 1^). The optimized temperature (*T*) and the reaction time (*t*) are 200 °C and 2 h. SA-CPDs is the brightest red CPDs with a high QYs of 21.4% (Additional file 1: Figure S3b). There are two reasons why H_2_SO_4_-assisted carbon dots have a better quality compared to HCl-, HClO_4_-, and HNO_3_-assisted ones (published in our previous work [25]). First, H_2_SO_4_ is a non-volatile strong acid which maintains its acidity in a high temperature and high pressure reaction solution. Second, H_2_SO_4_-assisted system is the only one that can form ammonium salt precipitation in precursor, and the precipitates release the free reactants slowly, avoiding the formation of large particle polymer precipitation and further promoting the formation of high quality carbon dots. HA-CPDs and PA-CPDs are dark-red-brown thick solutions and emit dark red PL under 365 nm UV light irradiation, while the as-prepared SA-CPDs is bright-red transparent thin solution and emits bright red light (Additional file 1: Figure S3c). After being purified by washing, concentration, filtering, and evaporation, dark-red-brown powders of SA-CPDs (Additional file 1: Figure S3d) were obtained with the product yield of 16.5%. The powders can be re-dissolved in water and the solution emits bright and red fluorescence (Additional file 1: Figure S3e).

### TEM characterization and FT-IR analysis

**Fig. 1:**
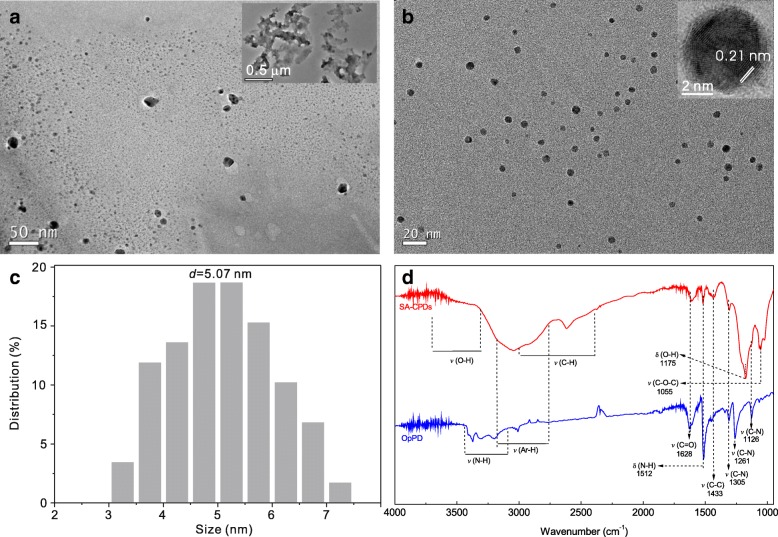
TEM images of sample OpPD (without H_2_SO_4_) (**a**) and SA-CPDs (**b**). **c** The size distribution of SA-CPDs. **d** The FT-IR spectra of sample OpPD and red CPDs. Sample OpPD and SA-CPDs were prepared from *p*-PD aqueous solution without and with H_2_SO_4_-assisted

TEM images of sample OpPD (without H_2_SO_4_ ) and SA-CPDs (with H_2_SO_4_) were shown in Fig. 1. Sample OpPD is composed of fragments (oligomers, Fig. 1a) and polymers (Fig. 1a insert). SA-CPDs are monodispersed CPDs with the average size of ~ 5 nm (Fig. 1c). It exhibits well-resolved lattice fingers with a space of ~ 0.21 nm (Fig. 1b insert), corresponding to the (100) in-plane lattice spacing of graphene [31, 32].

Surface states of CPDs may affect the optical properties. The surface chemical groups of sample OpPD and SA-CPDs were characterized by FT-IR spectra (Fig. 1d). Two samples have several similar groups, such as Ar-H (2700–3200 cm^− 1^ [33], belongs to aromatic C-H stretching vibration), C-C (~ 1433 cm^− 1^, belongs to aromatic C-bone stretching vibration, reveals the presence of aromatic stretching vibrations characteristic to benzenoid units) [34], and C=O (1628 cm^− 1^, belongs to the –COOH groups). Compared with sample OpPD, the new groups, such as O-H (3300–3700 cm^− 1^ and 1175 cm^− 1^ belong to the –COOH groups), C-O-C (1055 cm^− 1^, exist in esters), and C-H (2400–3000 cm^− 1^ belongs to alkyl radicals formed in ring-opening reaction), are found in the CPDs, while the –NH_2_ or –NH– related groups such as N-H (3100–3300 cm^− 1^ and 1512 cm^− 1^ and C-N (1126, 1261, and 1305 cm^− 1^ belong to the free –NH_2_ or –NH– groups from *p*-PD precursor, are weaken or disappeared. The existence of –OH or –COOH groups indicates that the oxidation degree of the surface of SA-CPDs (with H_2_SO_4_ adding) is higher than that of sample OpPD (without acid addition).

### Proposed Mechanism for the Formation of CDs

**Fig. 2:**
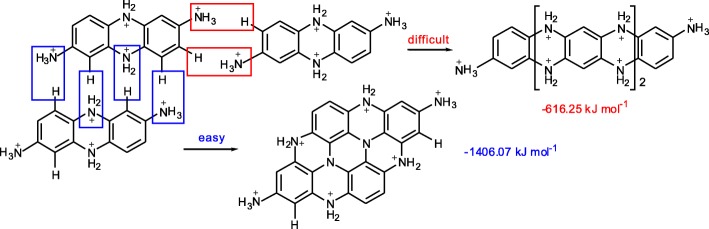
Formation energies of longitudinal and transverse growths

Formation energies were calculated using the Gaussian 09 program package. After being protonated by acid-assisting, bi-polymers may be polymerized in two ways that called longitudinal and transverse growth. The calculated formation energy of transverse growth (− 1406.07 kJ mol^− 1^) is significantly higher than that of longitudinal growth (− 616.25 kJ mol^− 1^). It shows that the fully protonated bi-polymers (pH 3, after excess H^+^ was added) tend to be polymerized in transverse way to form planar structure (Fig. 2). These planar structures were then self-assembled to form spherical CPDs.

### Optical properties

**Fig. 3:**
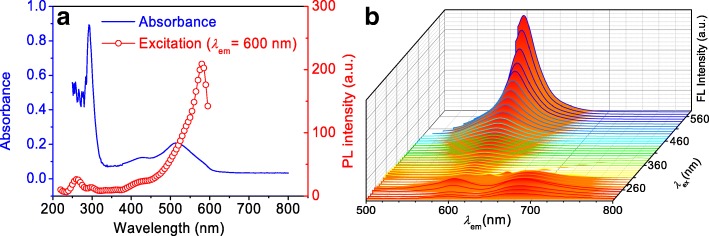
**a** UV-Vis absorption, excitation (peak at 600 nm), and **b** emission (excited 220–580 nm) spectra of SA-CPDs

Although being prepared by the assistance of different acids, all CPDs have the similar optical properties [25]. For the UV-Vis spectra of SA-CPDs aqueous solution (Fig. 3a), the absorbance peak located at ~ 290 nm is associated to the transitions in benzene ring, and the peaks located at 430 nm and 510 nm could be assigned to π-π* transition of substituted phenazine conjugated to the lone electron pairs on the adjacent amine group and the electron transition from the benzenoid ring to the quinoid rings, respectively [32]. The excitation curve describes a wide and a gradual upward trend at visible region, and the maximum excitation peak (~ 580 nm) is close to the emission peak (~ 600 nm). The CPDs emit at red light zone (600–700 nm) when they are excited from 220 nm to 310 nm, while emit at orange light (~ 600 nm) when excited from 310 nm to 580 nm (Fig. 3b). It is worth noting that the fluorescence of this kind of red-emitting CPDs is excitation-wavelength-independent [22, 35].

### Cellular Imaging

**Fig. 4:**
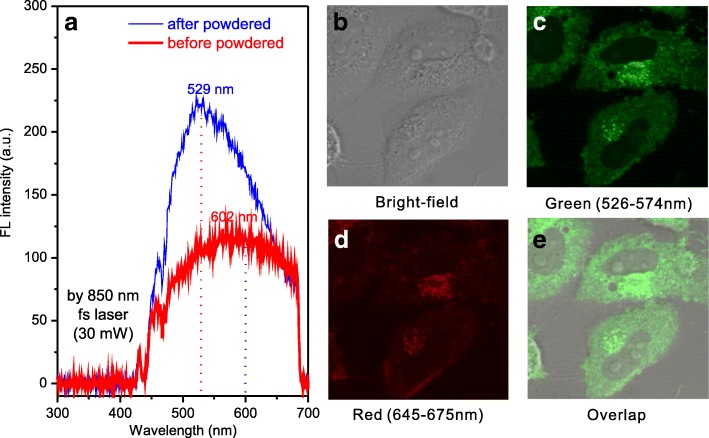
Two-photon photoluminescence spectra of SA-CPDs (**a**) and confocal fluorescence microscopy images of HeLa cells treated with SA-CPDs excited by 850 nm, 30 mW femtosecond pulse laser (**b**–**e**)

Two-photon photoluminescence properties of SA-CPDs before and after the powdering process are shown in Fig. 4a. There is a blue shift from 602 nm (before the powdering process) to 529 nm (after the powdering process) at the same excitation wavelength of 850 nm by femtosecond pulse laser (30 mW). The PL intensity was increased after the powdering process.

SA-CPDs powders were re-dissolved in PBS (1X) and was applied in HeLa cells imaging using confocal fluorescence microscopy and 850 nm femtosecond pulse laser (30 mW) (see Fig. 4b–e). After cultured with HeLa cells for 12 h, SA-CPDs were swallowed by HeLa cells, and CPDs entered cytoplasm. The FL intensity of red channel (645–675 nm) is weak while the green channel (526–574 nm) is bright, it is consistent with the blue shift in the powdering process.

## Conclusions

A facile method of acid-assisted hydrothermal route to prepare carbon dots and the application of bio-imaging were reported. Within H_2_SO_4_-, HCl- and HClO_4_-assisted systems, SA-CPDs prepared from H_2_SO_4_-assisted system is the brightest CPDs with the average size of ~ 5 nm, the QYs of 21.4%, and the product yield of 16.5%. SA-CPDs aqueous solution emits at 600 nm when excited by light from 300 to 580 nm. The emission wavelength is excitation-wavelength-independent. In addition, SA-CPDs have two-photon photoluminescence properties emitting at 602 nm when excited by 850 nm femtosecond pulse laser (30 mW). The method has also been utilized in imaging for HeLa cells and has the potential in, e.g., bio-imaging applications.

## Additional file

Additional file 1: **Figure S1.** Photos of CPDs samples under UV light (365 nm). **Figure S2.** The optimization process for HA-CPDs preparation with HCl-assisted *p*-PD system. **Figure S3.** (a) The suitable *c* (*p*-PD) range for the preparation of red CPDs at the optimized *c* (acid):*c* (*p*-PD) ratio. (b) QYs data and the intensity plots as a function of absorbance for the as-prepared CPDs excited at 365 nm. Samples in (a) were excited at 365 nm. (c) Photographs of all as-prepared and diluted C-dots samples under daylight and 365 nm UV light. (d) SA-CPDs powders and (e) the re-dissolved solution. (DOC 1178 kb)

Additional file 2: **Sheet 1.** Raw data of the size distribution. **Sheet 2.** Raw data of FT-IR. **Sheet 3.** Raw data of QYs. **Sheet 4.** Raw data of UV-Vis absorption and excitation. **Sheet 5.** Raw data of emission spectra. **Sheet 6.** Raw data of two-photon photoluminescence spectra. (XLSX 216 kb)

## Abbreviations

CDs: Carbon dots
CPDs: Carbonized polymer dots
HC-CPDs: Carbon Dots were prepared from *p*-PD with HCl-assisted system
PA-CPDs: Carbon Dots were prepared from *p*-PD with HClO_4_-assisted system
*p*-PD: *P*-phenylenediamine
QYs: Quantum Yields
SA-CPDs: Carbon Dots were prepared from *p*-PD with H_2_SO_4_-assisted system
