# Supplementary material for: Sulfuric Acid Assisted Preparation of Red-Emitting Carbonized Polymer Dots and the Application of Bio-Imaging
Source: Nanoscale Res Lett. 2018 Sep 10;13:272. doi: 10.1186/s11671-018-2657-4 (PMC6134856; doi:10.1186/s11671-018-2657-4)
Supplement: Supplementary file 1 — Figure S1. Photos of CPDs samples under UV light (365 nm). Figure S2. The optimization process for HA-CPDs preparation with HCl-assisted p-PD system. Figure S3. (a) The suitable c (p-PD) range for the preparation of red CPDs at the optimized c (acid):c (p-PD) ratio. (b) QYs data and the intensity plots as a function of absorbance for the as-prepared CPDs excited at 365 nm. Samples in (a) were excited at 365 nm. (c) Photographs of all as-prepared and diluted C-dots samples under daylight and 365 nm UV light. (d) SA-CPDs powders and (e) the re-dissolved solution. (DOC 1178 kb) [file 11671_2018_2657_MOESM1_ESM.doc]

Additional file 1

**Sulfuric Acid Assisted Preparation of Red-emitting Carbonized Polymer Dots and the Application of Bio-imaging**

Chunlin Tan^1^, Chao Zhou^1^, Xingyun Peng^1^, Huozhen Zhi^3^, Dan Wang^4^, Qiuqiang Zhan^1,*^ and Sailing He^1,2,*^

1.Centre for Optical and Electromagnetic Research, Guangdong Provincial Key Laboratory of Optical Information Materials and Technology, South China Academy of Advanced Optoelectronics, South China Normal University, Guangzhou 510006, China.

2.JORCEP, Department of Electromagnetic Engineering, Royal Institute of Technology, 10044 Stockholm, Sweden.

3.Engineering Research Center of MTEES (Ministry of Education), School of Chemistry and Environment, South China Normal University, Guangzhou 510006, China.

4.State Key Laboratory of Organic-Inorganic Composites, Beijing University of Chemical Technology, Beijing 100029, China.

† Corresponding authors: sailing@kth.se; qiuqiang.zhan@coer-scnu.org


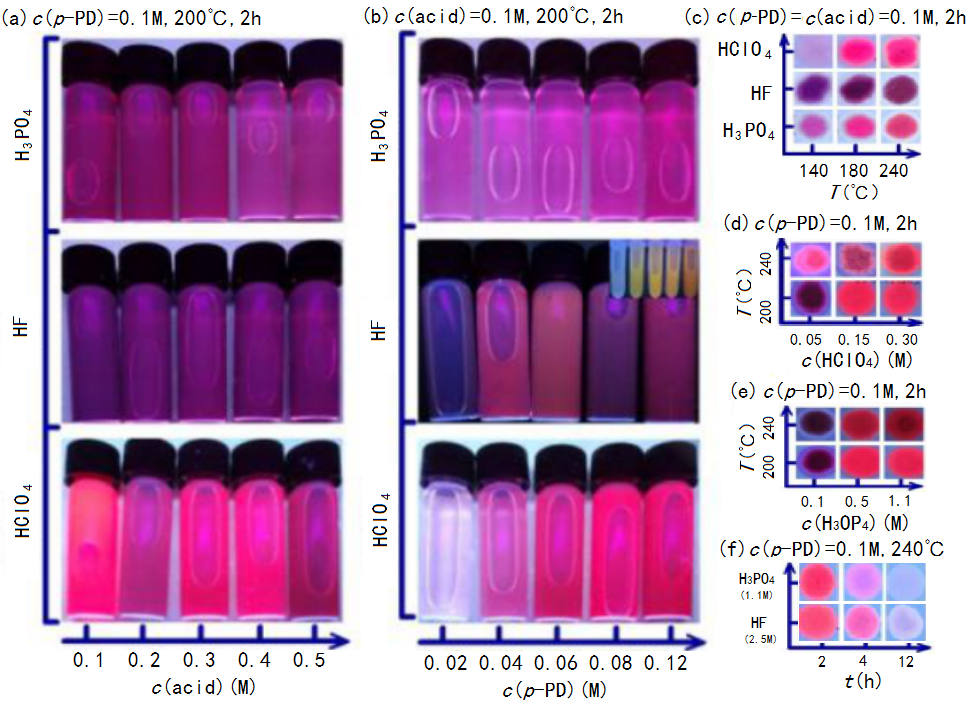


Figure S1 Photos of CPDs samples under UV light (365 nm)


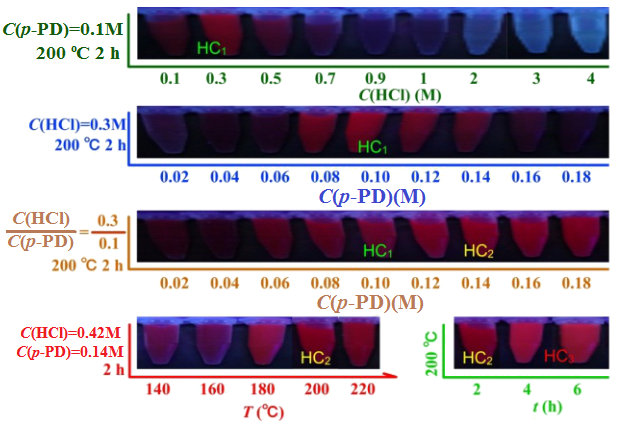


Figure S2 The optimization process for HA-CPDs preparation with HCl-assisted *p*-PD system


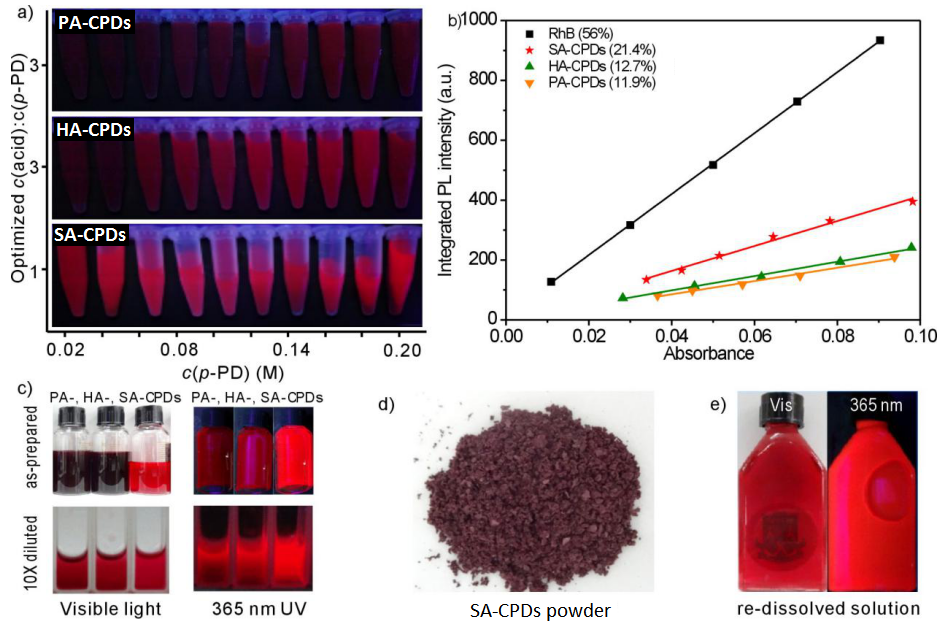


Fig. S3(a) The suitable *c*(*p*-PD) range for the preparation of red CPDs at the optimized *c*(acid):*c*(*p*-PD) ratio. (b) QYs data and the intensity plots as a function of absorbance for the as-prepared CPDs excited at 365 nm. Samples in (a) were excited at 365 nm. (c) Photographs of all as-prepared and diluted C-dots samples under daylight and 365 nm UV light. (d) SA-CPDs powders and (e) the re-dissolved solution.

**QYs Measurements**

The procedure of QYs measurements was based on the reference^[1]^ and our previous work^[2]^.

**First,** different concentrations of Rhodamine B (QYs=56% in ethanol) and the sample solutions (carbon dots in water) were respectively prepared, ensuring all the absorbance values A<0.1 at 365 nm.

**Second,** absorbance (by Lambda 950 UV/VIS/NIR Spectrometer, at 365 nm) and emission spectra (by F-2500 fluorescence spectrophotometer, emission range of 580-610 nm excited by 365 nm UV light) of the dye and sample solutions were tested respectively.

**Third,** find out all the absorbance *A* values and the corresponding integrated emission intensities *I*, draw a chart of *I* vs *A* points, and find the slope $k$ and $k^{'}$ by linear fitting of these points (see **Fig. S3b**).

**Last,** the QYs was calculated by

where and are the QYs of sample and reference dye,$I$and$I^{'}$are the integrated emission intensity of the sample and dye, $n$and$n^{'}$are the refractive indexes (1.33 for H_2_O and 1.36 for ethanol) of sample and dye.

Take $k$ and $k^{'}$ into the above formula and get (QYs).

**Reference**

[1] Grabolle M, Spieles M, Lesnyak V, Gaponik N, Eychmüller A, Reschgenger U. Anal Chem 81(2009) 6285-6294.

[2]Tan CL, Su XY, Zhou C, Wang BJ, Zhan QQ, He SL. RSC Adv 7(2017) 40952-40956.
